# Long-term trastuzumab (Herceptin®) treatment in a continuation study of patients with HER2-positive breast cancer or HER2-positive gastric cancer

**DOI:** 10.1186/s12885-018-4183-2

**Published:** 2018-03-15

**Authors:** Volkmar Müller, Michael Clemens, Jacek Jassem, Nedal Al-Sakaff, Petra Auclair, Eveline Nüesch, Debbie Holloway, Mona Shing, Yung-Jue Bang

**Affiliations:** 1 Department of Gynecology, University Medical Center Hamburg-Eppendorf, Martinistraße 52, 20246 Hamburg, Germany; 2 Innere Medizin I, Klinikum Mutterhaus der Borromäerinnen gGmbH, Feldstraße 16, 54290 Trier, Germany; 3 Department of Oncology and Radiotherapy, Medical University of Gdańsk, M. Skłodowskiej-Curie 3a, 80-210 Gdańsk, Poland; 4 F. Hoffmann-La Roche Ltd, Grenzacherstrasse 124, 4070 Basel, Switzerland; 5 Genentech, Inc., Global Pharma Development, 1 DNA Way, South San Francisco, CA 94080 USA; 6 Department of Internal Medicine, Seoul National University College of Medicine, 101 Daehak-ro, Jongno-gu, Seoul 03080 Republic of Korea; 7 Current affiliation: CaritasKlinikum Saarbrücken, St. Theresia, Rheinstraße 2, D-66113 Saarbrücken, Germany

**Keywords:** Herceptin, Trastuzumab, Breast cancer, Gastric cancer, HER2-positive

## Abstract

**Background:**

Trastuzumab (Herceptin® [H]) is the standard of care for HER2-positive locally advanced/metastatic breast cancer and gastric/gastroesophageal junction (GEJ) cancer. However, there is a paucity of data available on long-term H treatment of patients. The Rollover Protocol (ROP) Study was conducted to report safety data for patients with HER2-positive locally advanced/metastatic breast and gastric/GEJ cancer who have received long-term H therapy (≥ 5 years and ≥ 3 years for breast and gastric/GEJ cancer, respectively).

**Methods:**

The ROP Study was a single-arm, multicenter, international continuation trial of H in patients who had previously completed a global Roche-sponsored trial with H therapy, had stable disease, and were receiving H at the end of the lead-in trial. Patients with chronic heart failure during the lead-in trial could be included following a risk–benefit analysis. The primary objectives were to provide H therapy to patients with HER2-overexpressing locally advanced/metastatic breast or gastric/GEJ cancer at the end of the lead-in study, and to follow the long-term outcomes and long-term overall safety in these patients.

**Results:**

Twenty-five of 69 patients enrolled in the ROP Study received long-term H therapy (19 breast cancer and 6 gastric/GEJ cancer). The median duration of H treatment for patients with breast cancer was 8 years 7 months, and 5 years 2 months for patients with gastric/GEJ cancer. The cardiac status of the patients remained stable over time, with no serious cardiac adverse events or marked changes in left ventricular ejection fraction (LVEF). The median overall worst LVEF measurement was 57.0%, and no patients experienced an LVEF of < 45% (range 47–63%). There were no serious adverse events related to study treatment.

**Conclusions:**

These results suggest that H has an acceptable safety profile and was well tolerated in patients who received long-term H therapy (≥ 5 years and ≥ 3 years for breast and gastric/GEJ cancer, respectively). Further investigation and reporting of long-term H therapy would be valuable.

**Trial registration:**

This study was retrospectively registered on March 24, 2016 with Clinicaltrials.gov, number NCT02721641.

**Electronic supplementary material:**

The online version of this article (10.1186/s12885-018-4183-2) contains supplementary material, which is available to authorized users.

## Background

Trastuzumab (Herceptin® [H]; F. Hoffmann-La Roche Ltd, Basel, Switzerland) was first approved in 1998 in the United States, and first approved in 2000 in Europe, for the treatment of HER2-positive metastatic breast cancer; H has since been approved in more than 125 countries worldwide, which has led to establishing a new standard of care treatment for HER2-positive disease [1–5]. Despite the number of patients treated with H worldwide, there is a paucity of data reported on long-term H treatment. This Rollover Protocol (ROP) Study was initiated in 1998 to allow patients with HER2-positive metastatic breast cancer outside of the US, who had previously participated in Roche-sponsored HER2-positive breast cancer studies, to continue H therapy until disease progression.

H was approved in 2010 in both Europe and in the US for the treatment of HER2-positive locally advanced or metastatic gastric or gastroesophageal junction (GEJ) adenocarcinoma, and the ROP Study was expanded to provide H therapy until disease progression to patients who had completed any Roche-sponsored HER2-positive gastric/GEJ cancer studies.

The ROP Study was conducted to report safety data for patients with HER2-positive locally advanced/metastatic breast cancer and HER2-positive locally advanced gastric/GEJ cancer who have received long-term H therapy, defined as ≥ 5 years of H therapy for patients with HER2-positive locally advanced/metastatic breast cancer and ≥ 3 years of H therapy for patients with HER2-positive locally advanced gastric/GEJ cancer.

## Methods

### Study design

The ROP Study was a single-arm, multicenter, international continuation trial of H in participants who had previously completed a global Roche-sponsored trial with H therapy. The ROP Study was performed in accordance with the guidelines for Good Clinical Practice and the Declaration of Helsinki. Approval for the study protocol and any accompanying information provided to patients was obtained from ethics committees at each site (see Additional file 1). The ROP Study is registered on Clinicaltrials.gov, number NCT02721641.

### Study objectives

The ROP Study’s primary objectives were to provide H therapy to patients with HER2-overexpressing locally advanced/metastatic breast or gastric/GEJ cancer who had completed any global Roche-sponsored H clinical study, and to follow the long-term outcomes and long-term overall safety in patients treated with H.

### Patients

Patients were eligible to participate in the ROP Study if they had stable disease, and had their termination data, including tumor assessment and laboratory data, recorded on the Case Report Form (CRF) while receiving H at the end of their H lead-in trial [1, 2, 6–12]. Patients had received long-term H therapy, defined as ≥ 5 years for patients with HER2-positive locally advanced/metastatic breast cancer and ≥ 3 years for patients with HER2-positive locally advanced gastric/GEJ cancer. Patients with signs of chronic heart failure during the lead-in trial could be included following a risk–benefit assessment by the investigator. Key exclusion criteria were pregnant or nursing women, women of childbearing potential unless using effective contraception, severe dyspnea requiring supplementary oxygen therapy, and severe uncontrolled systemic disease. Signed, informed consent was obtained from all patients.

### Study treatment

Patients previously treated with H therapy continued to receive intravenous H (H IV) therapy administered according to the standard H IV weekly (4 mg/kg loading dose followed by weekly 2 mg/kg maintenance doses starting a week after the loading dose) or 3-weekly (8 mg/kg loading dose followed by 3-weekly 6 mg/kg maintenance doses starting 3 weeks after the loading dose) schedule, at the discretion of the treating physician. H therapy was continued until disease progression, investigator-assessed lack of treatment benefit, unacceptable toxicity, patient decision, or patient death. H administration could be delayed in the event of cardiac toxicity following an algorithm based on left ventricular ejection fraction (LVEF) assessment, but no dose modifications were permitted. If delayed, H was reinitiated in accordance with the reloading guidelines. For patients whose dose was delayed by ≤ 7 days, a maintenance dose was administered as soon as possible and subsequent maintenance doses were continued according to the original schedule. For those whose dose was delayed by > 7 days, a loading dose of 8 mg/kg or 4 mg/kg (depending on the patient’s dosing schedule) was administered and then maintenance doses were reinitiated and administered at regular intervals from that point. In addition to H therapy, continuation of treatment with other anticancer drugs at the same dose and schedule as that received in the H lead-in trial was permitted, provided that the treatment was still ongoing at completion of the lead-in trial and that it was still considered beneficial for the patient.

### Assessments

Tumor assessments for disease progression were performed as clinically indicated and at the discretion of the investigator, and the date of disease progression was recorded in the CRF. Cardiac status was assessed using an echocardiogram or multigated acquisition scan as clinically indicated. Cardiac failure (New York Heart Association [NYHA] class II–IV) was reported as a serious adverse event (SAE), and was graded as per NCI-CTCAE version 3 criteria or NCI-CTC version 2 and the NYHA classification system. SAEs were reported per the ICH Guideline for Clinical Safety Data Management, Definitions and Standards for Expedited Reporting, Topic E2. Hematology and biochemistry laboratory assessments were performed as per the standard medical practice at each site. The date of, and reason for, study completion were recorded in the CRF.

### Statistical analyses

The current analysis is descriptive and safety data are summarized herein. Sample size calculation was not applicable due to the nature of the study, and all eligible patients were included. No formal hypothesis testing was performed.

## Results

### Patients

Sixty-nine patients were enrolled in the ROP Study at 38 medical centers in 17 countries. Of these, 25 patients enrolled in 17 medical centers in 11 countries had received long-term H therapy, defined as ≥ 5 years of H therapy for patients with HER2-positive locally advanced or metastatic breast cancer and ≥ 3 years of H therapy for patients with HER2-positive locally advanced or metastatic gastric/GEJ cancer. These patients were included in the ROP Study after completing one of the nine global Roche-sponsored H trials in Table 1. Their baseline patient demographics and clinical characteristics are shown in Table 2. The median age at baseline of all patients included in the current analysis was 55 years (range 38–75 years) and 80% of patients were female (Table 2). The 19 patients with HER2-positive locally advanced/metastatic breast cancer were female and had a median age of 53 years (range 38–75 years). The remaining six patients (five males and one female) had HER2-positive locally advanced/metastatic gastric/GEJ cancer and had a median age of 61 years (range 52–74 years).

**Table 1:** Summary of Roche-sponsored lead-in H trials for the ROP Study

Lead-in protocol ID number	Study design	Patient population	Total patients in lead-in study, N	Patients included in ROP Study, n (%)
BO16216	Anastrozole ± H	Breast cancer	208	4 (16.0)
BO18255	H + cisplatin + FU or X	Gastric cancer	594	6 (24.0)
H0648g	H + AC or pac	Breast cancer	469	1 (4.0)
H0649g	H monotherapy	Breast cancer	222	1 (4.0)
M77001	H + T	Breast cancer	188	2 (8.0)
M77003	H + EC	Breast cancer	120	6 (24.0)
MO16419	H + T + X	Breast cancer	225	3 (12.0)
WO16229	H monotherapy	Breast cancer	105	1 (4.0)
WO17229	H + T or pac	Breast cancer	46	1 (4.0)

Abbreviations: *A* anthracycline, *C* cyclophosphamide, *E* epirubicin, *H* trastuzumab (Herceptin®), *FU* fluorouracil, *pac* paclitaxel, *ROP* Rollover Protocol, *T* docetaxel, *X* capecitabine

**Table 2:** Baseline patient demographics and clinical characteristics

	H
	*N* = 25
Median age (range)	55.0 (38–75)
Indication, n (%)	
Breast cancer	19 (76.0)
Gastric cancer	6 (24.0)
Sex, n (%)	
Female	20 (80.0)
Male	5 (20.0)
Country, n (%)	
Australia	1 (4.0)
Belgium	1 (4.0)
China	2 (8.0)
Germany	4 (16.0)
Hungary	1 (4.0)
Israel	2 (8.0)
Panama	1 (4.0)
Poland	2 (8.0)
Portugal	1 (4.0)
Republic of Korea	6 (24.0)
Russia	4 (16.0)

Abbreviation: *H* trastuzumab (Herceptin®)

At the end of the ROP Study, all patients included in this analysis had withdrawn from study treatment. Reasons for withdrawal included the occurrence of AEs (*n* = 1; 4%); insufficient therapeutic response, including disease progression (*n* = 6; 24%); switch to commercially available H therapy (*n* = 2; 8%); and refusal of treatment (*n* = 1; 4%). Other reasons for withdrawal were reported for 15 patients (60%), and included: study completion/closure (*n* = 5); sponsor request (*n* = 4); investigator decision (*n* = 3); switch to subcutaneous trastuzumab (Herceptin® SC, F. Hoffmann-La Roche Ltd) (*n* = 2); and agreement with the patient to discontinue treatment until further disease progression (*n* = 1) (Table 3).

**Table 3:** Reasons for study withdrawal

Reason for study withdrawal, n (%)	H
	*N* = 25
AE/intercurrent illness	1 (4.0)
Insufficient therapeutic response^a^	6 (24.0)
Switch to commercially available H treatment	2 (8.0)
Refusal of treatment	1 (4.0)
Other	15 (60.0)
Study completion/closure	5 (20.0)
Sponsor request	4 (16.0)
Investigator decision	3 (12.0)
Switch to H SC	2 (8.0)
Treatment discontinued until further disease progression	1 (4.0)

^a^Includes patients who experienced disease progression

Abbreviations: *AE* adverse event, *H* trastuzumab (Herceptin®), *H SC* subcutaneous trastuzumab (Herceptin® SC)

### Treatment duration

The median duration of H treatment in patients with breast cancer was 8 years and 7 months (3124 days), with a range of 5 years and 4 months to 13 years and 11 months (1940–5068 days). For patients with gastric/GEJ cancer, the median duration of H treatment was 5 years and 2 months (1886 days) with a range of 3 years and 4 months to 7 years and 4 months (1214–2677 days).

### Safety

**Fig. 1:**
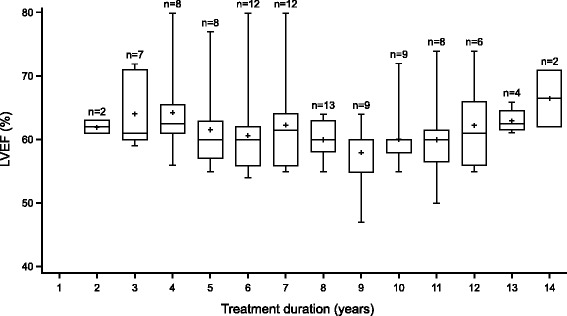
Worst left ventricular ejection fraction (LVEF) for each year of treatment. N is the number of patients with an LVEF assessment on study within the year on H. In cases of multiple LVEF assessments per patient within the same year, the lowest value is reported. Abbreviations: *H* trastuzumab (Herceptin®), *LVEF* left ventricular ejection fraction

Figure 1 shows the worst LVEF for each year of treatment. LVEF measurements were available for 20 of the 25 patients included in these analyses. The cardiac status of these patients remained stable over time, with no marked changes in LVEF. The median overall worst LVEF measurement was 57.0% and no patients experienced an LVEF of < 45% (range 47–63%).

In total, seven of the 25 patients (28.0%) reported SAEs (Table 4), none of which was related to study treatment. Reported SAEs were pain, septic arthritis, erysipelas, lung cancer, fibula fracture, urinary tract infection and a broken leg, which all occurred in one patient (4.0%) each (Table 4). There were no reports of any cardiac SAEs or SAEs that led to study withdrawal, and no patients died while on study treatment.

**Table 4:** Summary of serious adverse events (SAEs)

Preferred term, n (%)	H
	*N* = 25
All SAEs	7 (28.0)
Pain	1 (4.0)
Septic arthritis	1 (4.0)
Erysipelas	1 (4.0)
Lung cancer	1 (4.0)
Fibula fracture	1 (4.0)
Urinary tract infection	1 (4.0)
Broken leg	1 (4.0)

Abbreviation: *H* trastuzumab (Herceptin®)

## Discussion

An established body of clinical evidence has led to worldwide approvals of H as the standard of care for the treatment of patients with HER2-positive locally advanced/metastatic breast cancer and patients with HER2-positive locally advanced/metastatic gastric/GEJ cancer [4, 5]. Tolerability of H therapy in these patients has also been well established. As of September 2016, over 2.3 million patients have been treated with H therapy worldwide (data on file). However, despite this abundance of clinical experience, there is a paucity of data reported on long-term H treatment. Therefore, the results from this ROP Study of patients with long-term H therapy are of significant interest to the medical oncology community, as many physicians have ongoing queries about long-term management of patients with HER2-positive locally advanced/metastatic breast cancer or HER2-positive locally advanced/metastatic gastric/GEJ cancer who are being treated with H. To our knowledge, this is the longest study of patients receiving long-term H therapy for HER2-positive breast cancer or for HER2-positive gastric/GEJ cancer.

The results from this ROP Study suggest that long-term H therapy in accordance with clinical practice had an acceptable safety profile and was well tolerated. LVEF was stable in these patients, and only seven SAEs were reported in total, none of which was cardiac-related or resulted in withdrawal from the study.

The risk of cardiac AEs is a concern of H treatment. Earlier studies showed an increased incidence of cardiac dysfunction in patients treated with H, predominantly when H treatment was administered concurrently with chemotherapy drugs, and particularly in combination with anthracycline chemotherapy, in patients with HER2-positive metastatic breast cancer [2, 13]. In this ROP Study, however, the stable LVEF outcomes and the absence of cardiac SAEs suggest that long-term H therapy is feasible and without any additional cardiac safety risk, particularly with continued patient care and monitoring according to standard practice. Four patients had cardiac adverse events (three patients had tachycardia and one patient palpitation) reported from their respective lead-in study; however, three cases were resolved (one case resolution unknown) before the start of the ROP Study, and only one patient was enrolled from a lead-in study where patients had received H with anthracyclines. Therefore, the low level of cardiac SAEs observed in the ROP Study is due to enrollment of patients who have each demonstrated cardiac tolerability to H therapy in their prior lead-in studies, respectively. Other publications studying long-term H treatment in metastatic, locally advanced, or relapsed HER2-positive breast cancer also reported low risk of cardiac AEs and continued response [3, 14–16]; nevertheless, there remains a need for more information about patients with long-term H treatment, as reported by the ROP Study.

A limitation of these analyses is that enrolled patients had already been receiving H treatment previously, and hence may have had a lowered baseline risk of cardiac events and other SAEs in the ROP Study. In addition, patients selected for this analysis had to have received ≥ 5 years of H therapy for HER2-positive locally advanced/metastatic breast cancer or ≥ 3 years of H therapy for HER2-positive locally advanced/metastatic gastric/GEJ cancer. This selection means that the ROP Study population may not be representative of all patients with breast cancer or gastric/GEJ cancer who are treated with H. Therefore, the safety risks reported here may be underestimated in comparison with the general population, who may have to stop H treatment early due to AEs or disease progression. Underestimation of the safety risks may also be a result of underreporting of SAEs or missing LVEF measurements, although it is unlikely that relevant, serious cardiac AEs or significant LVEF decreases were missed in patients with long-term H therapy in the ROP Study, given that the patients were monitored regularly. Other limitations include differences in the lead-in protocols, in study center practices, and in rollover treatment regimens, and small patient numbers, which may limit comparison between patients. Finally, there is a paucity of additional information, such as tissue and serum biomarkers, on patients who performed well on H therapy and were enrolled into the ROP Study that may predict long-term benefits of H therapy.

## Conclusions

In conclusion, the results of the ROP Study suggest that in patients who received long-term H therapy for HER2-positive disease (≥ 5 years and ≥ 3 years for patients with HER2-positive locally advanced/metastatic breast and gastric/GEJ cancer, respectively), treatment with H had an acceptable safety profile and was well tolerated; further investigation and reporting of long-term H therapy would be valuable.

## Additional file

Additional file 1: **Table S1.** Institutions providing ethics approval for the ROP Study. (PDF 40 kb)

## Abbreviations

AE: Adverse event
CRF: Case report form
GEJ: Gastroesophageal junction
H: Trastuzumab
HER2: Human epidermal growth factor receptor 2
IV: Intravenous
LVEF: Left ventricular ejection fraction
ROP: Rollover protocol
SAE: Serious adverse event
SC: Subcutaneous
