# Supplementary material for: Long-term trastuzumab (Herceptin®) treatment in a continuation study of patients with HER2-positive breast cancer or HER2-positive gastric cancer
Source: BMC Cancer. 2018 Mar 15;18:295. doi: 10.1186/s12885-018-4183-2 (PMC5856394; doi:10.1186/s12885-018-4183-2)
Supplement: Supplementary file 1 — Table S1. Institutions providing ethics approval for the ROP Study. (PDF 40 kb) [file 12885_2018_4183_MOESM1_ESM.pdf]

Ethics approvals

The ROP study received ethics approvals from the institutions listed in Table S1.

Table S1: Institutions providing ethics approval for the ROP study

Lead investigator	Institution
Sergei Tjulandin	E.C. of FSBI Russian Oncology Research Center named after N.N. Blokhin of RAMS, Kashirskoye Shosse, 24, 115478, Moscow, Russian Federation
Vladimir Moiseyenko	E.C. of Research Oncology Institute named after N.N. Petrov of Ministry of Health and Social Development, Poselok Pesochny, Leningradskaya ulitsa 68, 189646, St. Petersburg, Russian Federation
Juan Carlos Alcedo	Comité Nacional de Bioética Inst. Conmemorativo Gorgas, Avenida Justo Arosamena y Calle 35, Inst. Conmemorativo Gorgas de Estudio de la Salud, 0816-02593, Panama
Ingo Bauerfeind	Ethikkommission der Medizinischen Fakultät, der Ludwig-Maximilians-Universität München, Pettenkoferstr. 8a, 80336, Munich, Germany
Volkmar Müller	EK Hamburg ÄK, Weidestr. 122 b, 22083, Hamburg, Germany
Michael Clemens	EK Rheinland-Pfalz LÄK, Deutschhausplatz 3, 55116, Mainz, Germany
Jacek Jassem	Niezalezna KB d/s Badan Naukowych przy AM w Gdańsk, ul. M. Skłodowskiej-Curie 3a, 80-210, Gdańsk, Poland
Sergei Primushko	E.C. of Republic Clinical Oncological Hospital, 102, Lenin str., 426009, Izhevsk, Russian Federation
Bella Kaufman	Helsinki Committee – Sheba Tel Hashomer, Sheba M.C., Ramat-Gan, Israel
Datong Chu	Local E.C. of Chinese Academy of Medical Science; Cancer Inst. & Hospital, Number 17, Panjiayuan South Road, 100021, Beijing, China
Yung-Jue Bang	Seoul National University Hospital Institutional Review Board, 101, Daehak-ro, Jongno-gu, 110-744, Seoul, Republic of Korea
Min Hee Ryu	Asan Medical Center Ethics Committee, 88, Olympic-ro 43-gil, Songpa-gu, 138-736, Seoul, Republic of Korea
Hyun Cheol Chung	Yonsei University Severance Hospital Institutional Review Board, Yonsei-ro 50, Seodaemun-Gu, 120-752, Seoul, Republic of Korea
Dominique Genre	CPP Sud-Méditerrannée I, Institut Paoli Calmettes, 232 Boulevard Sainte Marguerite BP156, 13273, Marseille, France
Keunwook Lee	Seoul National University Bundang Hospital Institutional Review Board, 82, Gumi-ro 173 Beon-Gil, Bundang-Gu, 463-707, Seongnam-Si, Gyeonggi-Do, Republic of Korea
Lin Shen	The Ethics Committee of Beijing Cancer Hospital, No.52 Fucheng Road, Haidian District, 100036, Beijing, China
Luis Miguel Zetina Toache	Comité de Ética Independiente Zugueme, 3a Calle 11-36, Zona 15, 01015, Guatemala

Guenther Bastert	EK Heidelberg, Alte Glockengießerei 11/1, 69115, Heidelberg, Germany
Michael Untch	Ethikkommission der Medizinischen Fakultät, der Ludwig-Maximilians-Universität München, Pettenkoferstr. 8a, 80336, Munich, Germany
Gerhard Schaller	Ethikkommission Charité – Universitätsmedizin Berlin, Chariteplatz 1, 10117, Berlin, Germany
Thomas Kraus	EK der Medizinischen Fakultät der Georg-August-Universität Göttingen, Robert-Koch-Str. 40, 37075, Göttingen, Germany
Klaus Diedrich	EK Lübeck, Ratzeburger Allee 160, 23538, Lübeck, Germany
Wolfgang Eiermann	EK Bayern LAK, Muhlbaurstr. 16, 81677, Munich, Germany
Rick Abraham	Royal Brisbane & Women’s Hosp Health Svc-Human Research, Level 7, Block 7 Butterfield Street, 4029, Herston, Queensland, Australia
John Stewart	Hunter New England Health HREC, Hunter New England Area Health Headquarters, Lookout Road, 2305, New Lambton, New South Wales, Australia
Vernon Harvey	Northern X Ethics Committee, 3rd Floor, Unisys Building, 650 Great South Rd, Penrose, 1061, Auckland, New Zealand
Josep Manel Baselga Torres	Comité Ético de Investigación Clínica, Hospital General Universitari Vall d’Hebron, Fundació Recerca Biomédica i Docència, 08035, Barcelona, Spain
Jacques De Greve	EC UZ Brussel, Laarbeeklaan, 101, 1090, Brussels, Belgium
Richard Bell	Barwon Health Research & Ethics Advisory Committee, The Geelong Hospital, Ryrie Street, 3220, Geelong, Victoria, Australia
David Grimes	Uniting HealthCare Human Research Ethics Committee, PO Box 499, 4066, Toowong, Queensland, Australia
Raymond Snyder	St Vincent Human Research Ethics Committee, 41 Victoria Rd, 3065, Fitzroy, Victoria, Australia
David Miles	Scotland A Research Ethics Committee; Secretariat, Waverley Gate, 2–4 Waterloo Place, Edinburgh, EH1 3EG, United Kingdom
António Moreira	CEIC – Comissão de Ética para Investigação Clínica, Parque da Saúde de Lisboa, Av. do Brasil, 53-Pav. 17-A 1749-004, Lisbon, Portugal
M. Jose Picon Cesar	CEIC Hospital General Universitario Alicante, Maestro Alonso 109, 03010, Alicante, Spain
Stephen Chan	Scotland A Research Ethics Committee; Secretariat, Waverly Gate, 2–4 Waterloo Place, Edinburgh, EH1 3EG, United Kingdom
Istvan Lang	Orszagoz Onkologiai Intezet Klinikai Onkol. Kozpont, Rath Gyorgy u. 7-9, H-1122, Budapest, Hungary
Janos Szanto	Medical Research Council, Ethics Committee for Clinical Pharmacology, Arany J. u. 6-8., 1051, Budapest, Hungary
Zoran Neskovic-Konstantinovic	Ethics Committee of Institute of Oncol. & Radiology, Pasterova 14, 11000, Belgrade, Serbia
